# Odonto-Chirurgical Society

**Published:** 1887-03

**Authors:** 


THE
AMERICAN JOURNAL
—OF—
DENTAL SCIENCE.

VOL. XX. THIRD SERIES—MARCH, 1887. NO. 11.

ARTICLE I.

### ODONTO-CHIRURGICAL SOCIETY.

At the meeting held on December 9th, a discussion arose upon the disease known as

### PYORRHŒA ALVEOLARIS.

The President said the disease, for some time termed Riggs' disease, and now better named Pyorrhœa Alveolaris, has long been known, but has only been much debated during the last twenty years by Riggs, Mills, Albrecht, Wedl, Salter, Tomes, Arkovy, Miller, Rehwinkel, and others more or less known in the scientific walks of dentistry. I do not propose to enter an opinion as to its pathology or etiology, nor yet to discuss the various methods of treatment or remedies advocated by these gentlemen, but will confine myself to a simple statement regarding the use of sulphur as a curative agent in this lesion. Whatever may be our opinion as to the part which tartar plays in this disease, I think we are all agreed that tartar being matter in the wrong place, it should, if present, be thoroughly removed, so as to give any subsequent treatment the best possible field for its recuperative action. This being done, I find that the bi-daily cleansing of the teeth and gums with a tooth-powder composed of flowers of sulphur and precipitated chalk, will very soon restore the parts to a healthy condition, the pus secretion will cease, and the teeth become firm in their sockets. Whether the tartar be the cause, or only a concomitant, or the pyorrhœa be owing to a perverted condition of the mucal secretions, sulphur will, and does, act beneficially.

Its therapeutic action is stimulating and antiseptic, and it is likewise a solvent of calculus. If it contains a little free sulphurous acid, which it frequently does, so much the better.

As I have said, it is stimulating, antiseptic, and a solvent of calculus, and it has this further, and what, I think, very great merit, viz., that it can be regularly and thoroughly applied by the patient. I have now been using it for over a twelvemonth, and have reason to be satisfied with its uniform success; and as I recommended it to the members of this Society in March last, I will be pleased to hear how it has succeeded with those who have given it a trial.

Dr. Smith said he had not been present at the last meeting, and his being called upon to make any remarks upon this interesting and somewhat important affection to-night was unexpected. In the first placc, he would say that he believed the mixed power of sulphur and magnesia, brought before the Society by Mr. Macleod, was, in all probability, destined to be of much service, not only in pyorrhœa alveolaris, but in other affections of the mouth. The *rationale* of its action seemed founded upon tangible principles, as there was no doubt that sulphur and its combinations had played a prominent part in the *role* of therapeutics as applied to similar affections. With reference to the pathological nature of pyorrhœa alveolaris, or Riggs' disease, two theories might be advanced—first, that it commenced from without; and second, that it commenced from within. Among those cases commencing from without might be classed such as originated in the alleged irritation caused by the deposit of tartar, or in inflammation of the gum and mucous membrane; and among those originating from within, the occurrence of alveolar caries would, in all probability, be found a common cause. Tartar and inflammation of the gum did not always give rise to the symptoms distinctive of pyorrhœa alveolaris, but caries of the alveolar walls invariably did so. The anatomy of the parts in a great measure accounted for this. It was admitted in surgery, that, as a general rule, pus seeks the nearcst and easiest mode of outlet, and if this were an affection of the gum and submucous tissue, it would manifest itself to a proportionately greater extent beyond the margin of the alveolar cavity than it very frequently does. But, in a large number of cases, the symptoms during its continuance, and the appearance and lesions of the alveolar walls after its existence, showed that these had been the structures principally concerned in the disease. This accorded with Dr. Smith's own observations in this affection, to which he had for long paid considerable attention; and also with the morbid appearances described by most writers on this disease. Dr. Smith had said that, *prima facie*, the disease commenced with, and consisted in, caries of the alveolar plate; and that the tartar deposit and inflammatory appearance of the gum, were possibly of a secondary nature. It might be argued that the alveolar plate was not altogether the kind of bone in which caries occurred; that necrosis and rapid separation of the dead part would be more likely to take place there than a long protracted ulcerative process. But this reasoning was here to be met in the local modifications of the bony tissue. Necrosis was, no doubt, the common affection met with in compact bone, and caries in the cancellous variety. This did not arise from the disease in its ultimate pathology being different in the two cases. In necrosis, the result of the inflammatory action and exudation was death of a piece of the bone by obliteration of the vessels contained in the Haversian canals, owing to the unyielding nature of the mass of compact bone surrounding them. In cancellous bone, room was afforded for dilatation of the vessels, so that complete stasis was not occasioned by the exudation. And in the very attenuated alveolar walls, the pressure outside the vessels would be comparatively diminished also, and might explain the more frequent existence of caries there.

The question whether this caries, or rather what is called rarifying ostitis, is a primary or secondary lesion, was difficult positively to decide. Cornil and Ranvier assert that it is secondary to fatty degeneration of the contents of the lacunæ, and subsequent inflammation and destruction of the trabeculæ in their vicinity, which then form so many centres of suppurative inflammation. While this is the opinion of one set of pathologists, others believed that inflammation precedes this fatty degeneration and may be of a simple scrofulous, tubercular, or syphilitic origin. At all events, the symptoms described by authors who have written on this disease would indicate, in the separation of the dento-alveolar structures, the channelling of the sockets and disappearance of their bony walls, that one form at least was probably due to alveolar caries.

It seemed very uncertain whether the deposition of tartar was not, in many cases, a result rather than a cause of the disease; the dark coloured and hard tartar was always found in much less quantities than the softer and white variety, and probably had been slowly deposited and stained in some manner due to this longer exposure of its surface. This dark tartar, too, was much more frequently found at the necks of the upper teeth than was the white variety. It was also found on the denuded fangs of teeth, which denudation, again, had probably been brought about by a form of alveolar caries, possibly without suppuration, as this was known to occur in other parts of the skeleton, and sometimes appeared more like ordinary absorption of the bone than any other form of disease.

Mr. Wilson said he quite agreed with what Dr. Smith had just said. The initiative was always periostitis, followed by disintegration of the alveoli, so that it might be the result of either local or constitutional causes.

He had lately advised the use of the powder recommended to their notice by Mr. Macleod, but could not yet say with what success.

Mr. Macgregor said he had used Mr. Macleod's prescription in one or two cases, and had found most marked and beneficial results following its use.

Mr. Durward spoke favourably of the sulphur powder, and said he had also found it a good powder for use where artificial teeth were worn above stumps.

Mr. C. W. Watson thought that if this mixture advocated by Mr. Macleod, used as a tooth powder, gave satisfactory results in the treatment of pyorrhœa alveolaris, it would be a great boon to us, as our patients would be able to take in hand their own treatment. In this disease, there is a pocketing of the gum round the teeth, softening and destruction of bone at alveolar margins, and an oozing out of a serous or sero-purulent secretion, which is laden with micro-organisms—bacteria, micrococci and bacilli (allied to algæ). This secretion seems to be derived from the connective tissue of the parts, and is thought to induce the same condition in adjacent healthy teeth by infection. He had tried to produce the disease in the dog by scraping its gum and spreading some of the secretion over it, but failed to get any result, though this might not be the case were a human subject inoculated.

The first step in the treatment of this disease is to scrape the softened bone and remove all the tartar, which, however, is not always present. In reference to this tartar, there is a variety found pretty far up on the root, of a dark greenish colour—especially in cases where there has been considerable destruction of the bone at the alveolar margin. This was, he thought, derived from the exudate and not from the saliva.

The same condition was observed, as a result of chronic alveolar abscess, where no sinus was present, and he had repeatedly found, on extracting such teeth, nodules of this dark tartar at or near the apex of the root, where no saliva could possibly reach.

The presence of micro-organisms in the secretion of the gum pockets must tend to keep up a great amount of irritation, if they are not the direct carriers of infection themselves, and it is, therefore, important to get rid of them as soon as possible.

They are divided into two groups—ærobian, those requiring oxygen for their development; and anærobian, those that do not require oxygen for their development. A mixture of hydrogen peroxide and hydrargyri perchloridi, injected repeatedly into the gum pockets, destroys both varieties, and this should be followed up with aromatic sulphuric acid, to cause granulation of diseased parts. This treatment, carried out every three or four days for several weeks, generally results in a speedy cure. It was to be hoped, however, this new cure would render this somewhat elaborate process unnecessary.

Mr. Amoore, in reference to Mr. Watson's recent remark, that the presence of tartar is not invariable in this disease, said he remembered Mr. C. S. Tomes citing a case in point during a discussion which bore largely upon this subject, at the International Medical Congress of 1881. It was the case of a patient aged 25, in which he extracted all the remaining teeth, and on many, which were distinctly affected, there was no trace of tartar. He had also heard the suggestion respecting the serous origin of the hard greenish variety once or twice before, and he thought he was right in referring it on one occasion to Mr. Coleman in one of his lectures. He (Mr. Amoore) had found it very high up on the roots of teeth, very commonly upon badly inflamed stumps, apparently beyond the reach of the saliva, though he could not recall having found any directly enclosed in an abscess sac.

---

The Third Ordinary Meeting of the Society was held on January 13th, the President, Mr. Bowman Macleod, in the chair.

The paper for the evening's consideration was entitled:

### A FEW NOTES ON ALVEOLAR HÆMORRHAGE.

BY JAMES M. NICOL, L. D. S., EDIN.

The object of the present paper is rather to act as the starting-point of a good and thorough discussion upon the subject of Alveolar Hæmorrhage, and to elicit the opinions of those members who, from long experience, have a right to offer them, than to contribute anything either new or startling. That it is an important question for the dental surgeon to know how to treat an obstinate case of hæmorrhage following upon tooth extraction, or any kindred operation in the mouth, all will admit; and such a Society as ours affords peculiar facilities for obtaining some definite and clear principles upon which to act in such a case.

The subject naturally divides itself into three parts, viz: —Varieties, Causes, and Treatment.

### I.—VARIETIES.

Our works on surgery divide hæmorrhage into three kinds—arterial, venous, and capillary, and in addition to this well-marked classification, various attempts have been made to classify according to the causes, and such terms as traumatic, spontaneous, active, passive, critical, periodical, have been used. These, however interesting they may be, so far as other hæmorrhages are concerned, do not enter into our calculations so far as the alveolar varieties may require our treatment.

For all practical purposes, alveolar hæmorrhages may be said to be included under the heads of Traumatic, Vicarious, and Constitutional.

(*a*) *Traumatic.*—In a sense, all alveolar hæmorrhages are traumatic, but it will, perhaps, be better to confine this term to the ordinary primary hæmorrhage following the extraction of one or more teeth. It is, as a rule, easy of control, and generally ceases of itself in a very short time. At the same time it may, from various causes, last so long that it becomes necessary to take means to stop it. If there be much inflammation of the surrounding tissues, primary hæmorrhage is likely to be very profuse and of some duration, owing to the vessels not contracting so readily as when in a healthy condition; or the profuse primary hæmorrhage may occur as the result of accident, such as a portion of the tuberosity of the upper jaw coming away in extraction of the wisdom tooth; or a lower wisdom tooth may have its roots embracing the inferior dental artery, and may cause its rupture in extraction. These are, however, rare occurrences, and speaking generally, the ordinary hæmorrhage following tooth extraction is not profuse or long-continued.

(*b*) *Vicarious Hæmorrhage.*—Probably the most frequent explanation of exceptionally profuse hæmorrhage is that it is vicarious of some other blood flow. The most common illustration of this is that of a woman having a tooth extracted at the menstrual period, when very frequently an alarming hæmorrhage will take place, and the menstrual flow be correspondingly diminished or absent altogether. Alveolar hæmorrhage may also be vicarious of epitaxis in full-blooded people who are subject to periodic attacks of bleeding at the nose. A little judicious inquiry will generally enable the operator to make up his mind upon a case of profuse primary hæmorrhage.

(*c*) *Constitutional.*—The hæmorrhages to which this term may be applied undoubtedly provide some of the gravest cases with which we have to deal. Fortunately they are so rare that many dentists pass through a long professional career without meeting with one. Certain constitutional conditions seem to predispose to hæmorrhage; an anæmatic condition, for instance, this being probably due to weakness of the contractile power in the vessels. Anything which tends to lower the system, as bad diet, or any chronic disease, is apt to produce a state of constitution favourable to hæmorrhage. In addition to all these, we have that special condition to which the name Hæmorrhagic Diathesis is applied, which is practically a confession of our complete ignorance as to its causes and treatment. What we do know about this state is; that it is often hereditary, that it is more frequent in males than females, but seems to be more often transmitted through the mother than the father, and that it is a congenital condition, usually manifesting itself first in early infancy; but as to its pathology the authorities differ completely. The symptoms include a marked tendency to hæmorrhage from very slight causes, or apparently from none at all, and in many cases swelling of the joints, especially the knee joint. That which makes a patient suffering from this condition such a serious responsibility is the fact that the primary hæmorrhage is often not at all profuse and shortly ceases. Then some time after, secondary hæmorrhage will commence, which may either be profuse from the socket of the alveolus, or may consist of a capillary oozing from the edges of the gum round the wound, this latter form being the most troublesome to stop. The case is often further complicated by the fact, that either through ignorance or carelessness, the dentist is not informed of the state of matters before the operation, or even immediately after it; and he may have extracted not one, but several teeth, thus increasing the gravity of the case a hundred-fold.

### II.—CAUSES OF HÆMORRHAGE.

These have practically been dealt with under the previous head; and need not be further discussed here, except that I would like to draw attention to a possible cause which I have not seen noticed hitherto, and that is alcoholic excess. My attention was drawn to this as a possible cause during the early part of last year. I had occasion to see a patient, a man of good position, who had ruined his constitution with drink; had passed through one attack of delirium tremens, and was then under treatment for the prostration following upon that attack. The first lower molar on the left side was very loose, and causing him a good deal of irritation, so it was extracted, and as the bleeding did not seem anything more than usual, no extra precaution was taken. The hæmorrhage, however, did not cease, but continued in the form of a slow dribble all that day and night; the patient then became alarmed, and it was necessary to pay three visits in one day and plug the alveolus firmly with lint soaked in Dr. Richardson's styptic colloid, each time, before the bleeding was finally checked. After a day's intermission it broke out again, but less profusely, and after plugging again in the same manner it finally ceased. One thing struck me very much in connection with this case, and that was the appearance of the gums, tongue, and surrounding tissues—they looked and felt perfectly disorganized and rotten, and I could quite believe that a scratch of the gums or tongue might easily set up another attack of bleeding. This coincides with the well-known fact that habitual drinkers make very bad subjects for accidents or surgical operations, their wounds as a rule not healing well. Of course it is impossible to build up any theory upon one case only, but I mention it in case anyone else may have noticed a similar one.

III.—TREATMENT.

Passing now to the all-important question of treatment upon which the welfare, and perhaps the life, of a patient may depend. In the case of simple profuse primary hæmorrhage, the treatment does not generally need to be of a very vigorous order. The application of a plug of lint saturated with Dr. Richardson's styptic colloid I have generally found sufficient, accompanied by rest in the recumbent position, with the head slightly raised. Where the hæmorrhage is vicarious it will generally be very profuse, but not of long duration; and the same simple measures will, as a rule, suffice to put an end to it. With regard to the more serious secondary hæmorrhage, the socket should be first syringed well out with warm water, then small strips of lint soaked in some styptic packed firmly down into it with a small ball-headed plugger; the lint is better used in small pieces, as you are thereby enabled to take the plug out piece-meal after the bleeding has been checked; whereas if you use one large strip of lint, the operation of loosening such a large plug is very liable to start hæmorrhage again. This plug of lint should be built up so as to project above the gum and then some form of compress must be used to press it firmly and evenly. A very simple and very efficacious one can be made by taking a piece of gutta-percha tube, about three-quarters of an inch in diameter, such as is used for call pipes, have the piece long enough to extend over one or two teeth in front and behind, slit it up one side and bend the edges apart, when it will be found to form a clumsy clamp, with a fair amount of spring in it; by means of a hot knife it can be pared to suit the shape of the jaw, and also gutta-percha can be built up on the top of it so as to meet any teeth in the opposite jaw. Let this clamp be lined with lint soaked in styptic colloid, and the edges of the gum round the plug carefully painted with a thick coating of the same styptic, then the clamp placed in position, and the patient directed to close the mouth until the teeth are about half-an-inch apart; this will allow of swallowing with a certain amount of ease, and will check any sucking of the parts, which is always to be prevented, as it only places matters where they were. As soon as the gutta-percha into which the opposing teeth bite has cooled sufficiently, the jaws should be carefully bound up and kept so for some days. Messrs. Ash & Sons' "Chin Appliance" for retracting the lower jaw will be found very useful for this purpose, and is rather more sightly than an ordinary bandage. When all hæmorrhage has ceased for some days, the compress may be removed, but it is generally wiser to leave the plug for a few days afterwards, as it generally loosens slightly in that time, and can be taken out with less risk of starting the hæmorrhage once more. Should there be very few teeth left in the mouth, or none at all, I should think the best plan would be to take models, and strike upper and lower plates, to which very strong spiral springs might be attached, and the plates lined in the manner indicated above. If the patient is only kind enough to let the dentist know beforehand the danger he is running, I believe that in the great majority of cases secondary hæmorrhage can be prevented altogether by plugging the socket immediately after the operation with strips of lint soaked in the styptic colloid. It seems as if very little would check the secondary hæmorrhage at the moment of commencing, but once it gets fairly started it is much more difficult to stop. I have found several patients who announced themselves as "bleeders" give very little trouble when treated in this way. I should certainly under no circumstances extract more than one tooth at a time for a "bleeder." The multiplication of wounds only increases the difficulty of stopping the hæmorrhage. With regard to styptics; as you will have gathered, I pin my faith to Dr. Richardson's styptic colloid. It has many advantages over perchloride of iron; the latter making a nasty mess of the mouth, and even causing inflammation. Properly applied, in combination with firm pressure, I have never known the styptic colloid to fail. With regard to other local measures, the actual cautery has been tried in several cases, but with scant and only temporary success; and there is always the risk of wounding the cheek or lip in applying it. Matico leaf, tannic acid, gallic acid, and turpentine have also been tried, sometimes with success, sometimes not. In one or two cases on record, everything that could be suggested has been tried without success, and the termination has been the death of the patient. Of such a case, my old master, the late Dr. Roberts, of Edinburgh, gave an account in his paper read before this Society some years ago, and the results of that case are to be seen in the Society's museum, in the shape of an ingenious compress which he invented for checking alveolar hæmorrhage. I well remember that the gist of his teaching to his pupils in this subject was styptics and pressure, and I am bound to say I have found his teaching correct. I have said nothing on the subject of constitutional treatment, as I consider that belongs to the medical man's department, and such a serious thing as a bad case of secondary hæmorrhage should always be treated in conjunction with the physician or surgeon. But I cannot leave the subject without referring briefly to the important point opened up by the case communicated to the Odontological Society by Mr. J. S. Turner, at one of the recent meetings, in which the patient had been prepared for the extraction by being put on a course of tincture of ergot and sulphuric acid for a week previous to the operation. It was an undoubted case of hæmorrhagic diathesis, as the patient had upon several previous occasions bled almost to death as the result of tooth extraction; on this occasion, however, although secondary hæmorrhage set in about twenty-four hours after the operation, it was of a very slight and unimportant character, and soon passed off. In this case Mr. Turner had the advantage of knowing beforehand the condition ot his patient, and under similar circumstances I should certainly see the patient's medical attendant, and consult with him as to the advisability of some similar course of preventive treatment; but as it often happens that the dentist knows nothing about the danger until the operation is over, it is imperative that he should have all his apparatus ready, and at hand, for stopping all hæmorrhage.

It will be found an excellent plan to fit up a small box or bag with all the most useful requisites for arresting hæmorrhage, so that if called out at uight, as has happened to the writer, you have nothing to do but lift your bag or box, and go.

One case of hæmorrhage, if it be a serious one, will be quite as much as any dental surgeon will ever wish to encounter; and after meeting with it, he will be much more likely to over-estimate the risks of prolonged bleeding than to under-estimate them.

In conclusion, I can only enforce, as the result of what little experience I have had, the great desirability of plugging the cavity at once after the extraction, before the patient has left the house, if there is any suspicion of liability to hæmorrhage. As I have pointed out, this will often prevent secondary hæmorrhage altogether, and it is the secondary hæmorrhage which is to be so much dreaded; besides, it also prevents the injudicious attempts which the patient and friends make to stop the bleeding before calling in help; which often consist of putting the patient in front of a roaring fire, wrapped up in blankets, and administering hot drinks and mouth washes; thus aggravating the very state of things which they want to stop; and, of course, the longer a case of hæmorrhage is allowed to go on before it is treated, the less likely is treatment to be successful.

In the discussion which ensued—

Mr. Wilson said, that in all ordinary cases (that is, where the blood was forming a clot) he found the mere plugging the alveolus with cotton wool soaked in Richardson's styptic, and then placing over the plug and alveolar margins a saddle of cork, thick enough to bring direct pressure to bear on both by the closure of the jaws, quite sufficient. The saddle was made to fit in as tightly as possible between the teeth on each side, so that none of the pressure was lost, which would be the case if the saddle, whether made of cork, gutta-percha, or other material, was so broad as to include these teeth.

He was thankful to say that of these cases, in which there was a want of coagulability, he had only met with one, the bleeding being set up by the patient (a boy) picking out a small morsel of a temporary root. Suspecting the patient of aggravating it by sucking, he covered a considerable surface with a gutta-percha-lined plate, and the case yielded to constitutional treatment. He decidedly objected to the use of perchloride of iron, or burnt alum, as styptics in the mouth.

Mr. Macgregor mentioned one or two cases which had occurred in his practice. About fourteen years ago, the late Dr. Angus Macdonald called him up about oue o'clock one morning, to see a patient of his who was suffering from a severe attack of hæmorrhage from the sockets of a lower molar and bicuspid tooth, which had been extracted on the preceding day. The doctor had, for four hours, been unsuccessfully endeavouring to arrest the flow of blood, using perchloride of iron as a styptic. Mr. Macgregor removed the clots of blood, and syringed the part thoroughly, and applied Richardson's styptic colloid. The first application was unsuccessful, but after applying it a second time, and using considerable force in packing the cotton steeped in the styptic down into the sockets, the hæmorrhage ceased. He waited for about an hour to assure himself that no recurrence was likely, and then left. The case was rather a serious one, as the patient was very weak and anæmic from the loss of blood, which had been considerable, and had it proved obstinate and recurred, a serious result was apprehended.

On another occasion, when putting in an artificial denture, he noticed that a loose root interfered with the adjustment of one of the clasps, and to remedy the matter, removed it with the point of an excavator. Two days afterwards, the patient returned with the blood flowing from this shallow socket. In the interim, she had been to a druggist, who had attempted to arrest it with perchloride of iron, until the mouth was perfectly blackened with it. The bleeding was stopped in a short time by a steady application of the styptic colloid.

In one instance, which occurred to him, the hæmorrhage recurred in the mouth of a patient after it had been arrested for one or two days. He had, when occasion required, used the saddle-shaped piece of cork to keep the plug in position, as referred to by Mr. Wilson, and found it very efficacious.

Mr. Finlayson mentioned several cases which had occurred in his practice, similar to those spoken of in Mr. Nicol's paper, particulars of which he gave to the meeting.

In treating hæmorrhage proceeding from extraction of teeth, he made sure that all clots were cleared out, these being, in his opinion, the main cause of the bleeding, as they were generally of a soft fibrinous character, containing fluid blood in their substance, and of a spongy nature, thus serving to keep the wound open. This having been accomplished, the edges of the gums were steadily and firmly pressed together with a slipping motion of the finger and thumb, and a previously prepared saddle of dry lint bound with floss silk, to prevent change of form or absorption, carefully adjusted to bring pressure to bear on the sides of the gums--more than on the top--the depth of saddle being so arranged that it came first in contact with the opposing teeth or gum. He had never plugged the socket.

In all cases, a chin bandage ought to be applied to prevent the lower jaw pressure from being removed for, at least, five or six hours—milk diet, quiet, a sitting posture, and coolness of surroundings being insisted on. Many cases of bleeding owed their origin to the administration of stimulants before or after the operation, and he had, for some years, adopted the rule of cautioning patients, either in hospital or private practice, to avoid the latter when unusual bleeding occurred in the surgery.

Mr. Finlayson mentioned the case of a boy, eight years of age, who had taken out one of his own teeth; the resultant hæmorrhage being so profuse and continuous as to defy all treatment. Those in attendance were expecting death; the pulse being almost imperceptible, and the countenance pale to a degree. In this case the bleeding stopped short of causing a fatal result, and the boy made a rapid recovery, being seen a few weeks afterwards running about apparently well, but with that delicate complexion peculiar to those of hæmorrhagic habit. The mother was a "bleeder" also.

Leech bites had sometimes proved troublesome, but, as a rule, the cleansing away of all soft stringy clots, and the application of moistened matico, or the lunar caustic point, with immediate and continued pressure, dry lint pads being used, had always proved sufficient to stay further bleeding.

A very good internal remedy, in cases of this sort, he had found to be an acidulated solution of sulphate of magnesia, which, given every hour, acted as a depressant and astringent.

Mr. Munro made a few remarks to the effect, that if in a case of alveolar hæmorrhage the blood was seen to issue in small jets from the socket, or there was reason to suspect the partial rupture of a small artery, it would be advisable, before applying the plug and pressure, to pass a small sharp instrument down into the socket and completely divide it, and thus give natural hæmostatics a fair chance by allowing the inner coats of the artery to retract within the sheath, and afford a better opportunity for the blood to coagulate.

Mr. Mackintosh had found the styptic colloid most valuable, and had also, in one or two instances, had occasion to tie up the jaw in the manner indicated by the previous speakers. He had also found water, as hot as it could be borne in the mouth, very effectual in arresting primary hæmorrhage.

The President said that the paper with which Mr. Nicol has favoured us is a good and interesting one is evinced by the hearty way in which its subject and premises have been discussed. Few practitioners of any length of practice but have had, at one time or other, considerable concern regarding the ultimate issue of some case of persistent or of secondary hæmorrhage. I fancy, however, if I may judge from my own experience, that there is seldom any cause for grave apprehension, the cases being few and far between which do not yield to careful and rational treatment. I would take exception to the general statement of Mr. Nicol, that where you have a predisposition to bleeding not more than one tooth should be extracted at a sitting. I find in practice, that the greater the number of teeth removed, the less proportionately is the hæmorrhage which follows, and in answer to the query, Why? I would illustrate it by analogy. Consider, for one moment, the teeth to represent so many tenements in a street, and that each house is supplied with water by branch pipes from the main. The total calibre of these branches is greater than the calibre of the main. Open one branch, the pressure is great; open two, and the pressure is reduced; open them all, and the pressure is reduced to the minimum. So with the blood-vessels, by opening more branches you reduce pressure and give every chance to the tonic and contractile powers of the vessels to close up upon the clot forming with the sheath, and making a firm and non-porous plug. The large clots, seen in bleeding cases, result from a too rapid coagulation, and are consequently porous and spongy. Hot water, as a styptic, is a very useful application, the heat of the water should be about 120° F., hotter will scald and destroy the tonicity of the vessels, as well as impair the integrity of the clot, while the action of water at, or about, 120° is that it expands the vessels, encourages coagulation, and the vessels being expanded during coagulation, upon cooling down to the normal temperature contracts and compresses the clot and renders it more dense. Cold water, on the other hand, being a capital solvent of blood, encourages bleeding; when cold is applied to a bleeding vessel or surface, it must be applied in a dry form. I use a very similar compress to that described by Mr. Wilson, using "Godiva" or "Stent" instead of cork. Within the modelled compress I place cotton wool, and super-saturate it with collodion, placing it in position, fixing the under jaw with a two-tailed bandage. This I sometimes precede with a lead and opium pill. I never plug the socket—firstly, because I obtain better results without it; and secondly, because the removal of the plug is apt to induce a recurrence of the hæmorrhage. As for Richardson's styptic, while admitting that it is an excellent styptic, I think better results are obtained by the use of *pure collodion*, the presence of the tannin either being inoperative, or if operative, it must be dissolved from out the collodion sheath, and render it less strong and valuable as an impervious covering.

I am glad that Mr. Munro has called attention to one cause of bleeding, perhaps more frequently attending tooth extraction than we are apt to admit, viz., the rupture of some small artery. The simple cure in such a case is, as Mr. Munro has pointed out, to take a fine-pointed lancet and cut the artery through; the mouths of the severed artery will then contract and the bleeding cease.

The thanks of the Society having been given to Mr. Nicol for his most admirable paper, the meeting adjourned.—*The London Dental Record.*

